# Ouida against the Physiologists

**Published:** 1891-10-03

**Authors:** 


### The Doctor's Armchair.

OUIDA AGAINST THE PHYSIOLOGISTS.

There is one thing the clever woman is endowed with which often makes her the admiration and envy of the writing man, and that is courage. Granted that the lady is experienced in the use of the pen, her natural intrepidity is to her a sort of Fortunatus' hat. However little she may know about a given subject, however much there may to be known, and however difficult the knowledge may be of acquisition, the writing woman, sublimely indifferent to the possible consequences which may ensue, rushes in with unblenching cheek, as if all knowledge were her own conquered province. Ouida, clever as she is, is no exception to this almost universal rule. It is as clear as noonday that she has no apprehension whatever of the temper of science, even in its most rudimentary condition. The world, in its scientific aspect, is a closed book to her. She belongs to a past generation. And yet the really scientific view of things is the true view, and it is the view to which all men, and women too, will have to come, if they would save their intellects alive. The unscientific is gradually becoming the uneducated. This, too, is as clear as the sun to the man who has an open eye for his own time.

Ouida is in arms against Jenner, Pasteur, and Koch, and still more against the great civilised states which try to make physiological knowledge effective for the general good. The State, she says, "in its strenuous endeavour to cure physical ills, does not heed what infamies it may sow broadcast in the spiritual fields of the mind and heart. It treats altruism as criminal when altruism means indifference to the contagion of any infectious malady. The precautions enjoined in any such malady, stripped bare of their pretences, really mean the naked selfishness of the *sauve qui peut*. The pole-axe used on the herd which has been in contact with another herd infected by pleuro-pneumonia, or anthrax, would be used on the human herd suffering from typhoid, or small pox, or yellow fever, or diphtheria, if the State had the courage to follow out its own teachings to their logical conclusions. Who shall say it will not be so used some day in the future, when increase of population shall have made mere numbers of trifling account, and the terrors excited by physiologists are of ungovernable force? We have gained little by the emancipation of human society from the tyranny of the Churches, if in its stead we substitute the tryanny of the State. One may as well be burned at the stake as compelled to submit to the prophylactic of Pasteur or the lymph of Koch."

This high flying lady probably has a magnificent contempt for plain facts and common sense. Very well; but common sense may congratulate itself when it is not tied by unbreakable chains to flighty rhetoricians, who are of as much real importance in the world, and no more, than the foam is to the sea out of which it is whipped by the wind. It would not concern us to remark at all upon what so inconsequent a physiological writer as Ouida has said about physiologists and the State application of health laws, were it not that many people attach an absurd importance to everything they see in print, and keep themselves in a constant state of readiness to be imposed upon by anybody who has a little more cleverness and a little more courage than themselves.

The State is anxious to cure bodily ills, says Ouida, and does not care at all what spiritual ills it produces in the process. Now of this there is not even a shadow of proof. The real facts are, that the State, in England at any rate, is anxious to cure both bodily and spiritual ills. But if we ask whether of the two sets of ills it has hitherto shown itself most anxious to cure, we shall find it is the spiritual and by no means the bodily. Let us apply a money test to the case. Even the flighty Ouida, we fancy, is practical enough to understand the value of a cheque. Our experience is that the most spiritual woman would rather have two guineas a column than one for her literary contributions to the illumination of the world. This is not said cynically, but for the purpose of convincing Ouida that plain practicality is sometimes worthy of a little consideration. To return to our money test. The State of Great Britain from the time of the Norman Conquest has spent certain sums of money upon religious establishments, secular education, and sanitary improvements. The question is—Which of these three sets of institutions—the religious, the educational, or the sanitary—has cost the State the largest sum? Now, we affirm with all possible boldness, that, during the last eight hundred years, that is, from the date of the Norman Conquest until now, where the State has spent one pound in sanitation, it has spent a thousand in education and a million in religion. It is not said that a single penny too much has been spent either on religion or on education, but it is affirmed that a State which has spent more millions upon religion than it has spent pounds upon sanitation, cannot justly be charged with being heedless "what infamies it may sow broadcast in the spiritual fields of the mind and heart."

It is true that the State has enforced compulsory vaccination, and has also passed the Contagious Diseases Act. Well, the State makes no claim to infallibility. In acting as it did on the historical occasions here mentioned, it simply followed the advice of those whom it believed to be most competent to give advice, and who at least knew more about the subjects dealt with than any other persons of their times. It can undo what it then established. In striving to promote the physical health of the people, the the State was as mindful of their spiritual health as of their bodily well-being. For the modern State happily knows this at least, that there can be little spiritual prosperity where material health and happiness are unknown. Ouida herself, it cannot be doubted, if she were to approach the subject from a different side, would make the very contentions here affirmed.

It is insinuated that physiology attempts to tyrannise over the people through their bodily fears, exactly as the Church of the middle ages ruled them through their spiritual terrors, and that the State is but too willing to play the game of the physiologists. "One may as well be burned at the stake," says Ouida, "as compelled to submit to the prophylactic of Pasteur, or the lymph of Koch." Quite so, Madame Ouida: but take a sniff at your smelling bottle, and calm your fears. If you were a man instead of a woman it might be advisable for you to put your fevered head under the pump, or even to wear an ice cap for two or three hours. So far from the State inclining to the physiologists, it deliberately, by the gloved hand of Sir Michael Hicks-Beach, gives them a slap in the face. Not only is there no tendency to increase the range of prophylactic inoculation, but there is a distinct set of the tide in the opposite direction. The State may very well multiply its efforts tenfold for the material well-being of its children, and still keep thousands of miles away from any injury to the spiritual parts of their nature. Compulsion on the lines of Jenner, Koch, and Pasteur will probably not in the future find favour among legislators; but sanitary science in its widest sense must be accepted as a religious creed by all those States who would do the best that is possible for the souls as well as the bodies of their people.

